# Furocoumarin Derivatives from Radix Angelicae Dahuricae and Their Effects on RXRα Transcriptional Regulation

**DOI:** 10.3390/molecules16086339

**Published:** 2011-07-26

**Authors:** Dong-Ping Liu, Qiang Luo, Guang-Hui Wang, Yang Xu, Xiao-Kun Zhang, Quan-Cheng Chen, Hai-Feng Chen

**Affiliations:** 1 School of Pharmaceutical Sciences, Xiamen University, Xiamen 361005, China; Email: liudongping333@126.com (Q.-P.L.); luoqiang20032004@yahoo.com.cn (Q.L.); guanghui@xmu.edu.cn (G.-H.W.); xu_yang@xmu.edu.cn (Y.X.); xzhang@sanfordburnham.org (X.-K.Z.); 2 Sanford-Burnham Medical Research Institute, La Jolla, CA 92037, USA

**Keywords:** Radix Angelicae Dahuricae, furocoumarins, nuclear receptor RXRα

## Abstract

A novel furocoumarin derivative named oxyalloimperatorin (**1**), together with seventeen furocoumarins **2–18** were isolated from the radix of *Angelica dahurica*. The chemical structure of new metabolite was characterized by analysis of IR, NMR, and HR-ESI-MS spectroscopic data. Among the isolated compounds, **13**, **16**, and **18** (each at 20 μM) could significantly promote the gene transcriptional function of nuclear receptor RXRα. While **7**–**9**, **13**, **14**, and the new structure **1** (each at 20 μM) showed significant reduction in RXRα gene transcriptional activities induced by 9-*cis*-retinoid acid. The findings indicated that these furocoumarin skeleton derivatives might hold beneficial effects on many intractable diseases, such as cancer and metabolic diseases, due to their potential activities on regulating the transcriptional activation function of RXRα.

## 1. Introduction

Radix Angelicae Dahuricae, the root of *Angelica dahurica* (Fisch. ex Hoffm) Benth, et Hook. f. var.formosana (Boiss.) Shan et Yuan, has been widely used as a traditional medicine in China for the treatment of toothache, headache, cough, asthma, coryza, *etc.* [1]. Previous phytochemical investigation revealed that the root of *A. dahurica* possesses various chemical composition including volatile oil, coumarins and glycosides. In them, coumarins were the most important major components with many remarkable activities, such as anti-inflammation [2], anti-bacteria [3], and lipogenetic inhibition [4]. In addition, many studies have been concerned about the anticancer effect of coumarins from *A. dahurica* [5,6,7,8]. 

The retinoid X receptor-α (RXRα) is a member of the nuclear receptor superfamily of ligand-activated transcription factors and an obligatory heterodimer partner for many nuclear receptors such as the peroxisome proliferator-actived receptor (PPAR), the retinoic acid receptor (RAR), and the liver X receptor (LXR) [9]. It plays key roles in various biological processes including cancer, diabetes, obesity, and atherosclerosis, and both agonist and antagonist of RXRα have been revealed to exert beneficial effects in such diseases [10,11,12]. In recent years, more and more studies have been focused on screening small molecules with regulatory function to RXRα from nature [13,14,15]. The specific aim of our present study was to identify novel naturally occurring metabolites with regulatory effects on RXRα gene transcriptional activation. Several furocoumarins were isolated from the radix of *A. dahuricae* and their transcriptional activities were examined by reporter gene assay.

## 2. Results and Discussion

Chromatography of the EtOAc-soluble extract of the Radix Angelicae Dahuricae produced a novel furocoumarin derivative **1**, together with seventeen furocoumarins **2-18**. The known compounds were identified as isoimperatorin (**2**) [16], cnidilin (**3**) [17], phellopterin (**4**) [18], bergapten (**5**) [18], imperatorin (**6**) [19], xanthotoxin (**7**) [5], alloimperatorin (**8**) [16], isooxypeucedanin (**9**) [20], isodemethylfuropinarine (**10**) [21], xanthotoxol (**11**) [16], 5-methoxy-8-hydroxypsoralen (**12**) [22], demethylfuropinarine (**13**) [23], apaensin (**14**) [24], pabulenol (**15**) [25], isobyakangelicin (**16**) [26], byakangelicol (**17**) [18] and oxypeucedanin hydrate (**18**) [17], respectively. Among them, **10** and **13** were isolated from *A. dahurica* for the first time.

**Figure 1:**
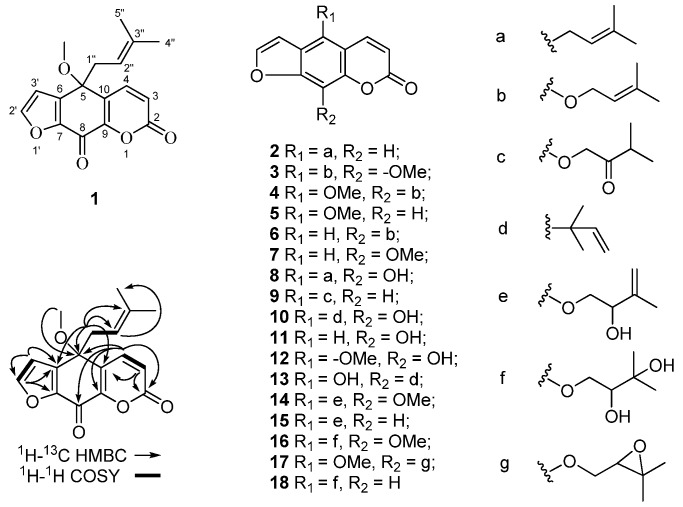
Chemical structures of compounds **1**–**18** from Radix Angelicae Dahuricae and the Key HMBC and ^1^H-^1^H COSY correlations of compound **1**.

Compound **1** was obtained as a white amorphous powder, [α]^22^_D_ +5 (*c* 0.4, MeOH). Its molecular formula, C_17_H_16_O_5_, was established by HR-ESI-MS with a mass of [M + Na]^+^ (*m*/*z* 323.0894, calcd. 323.0890). The IR spectrum of **1** showed characteristic absorptions of α,β-unsaturated lactone (ν_max_ 1738 cm^–1^), and α,β-unsaturated carbonyl (ν_max_ 1687 cm^–1^) groups. The ^1^H-NMR spectrum of **1** showed two methyl, one methoxyl, five olefinic, and one methylene proton. The ^13^C-NMR, and DEPT spectra of **1** displayed 18 carbons, including two methyls, one methoxyl, five olefinic methines, two ketone carbonyl, and five quarternary carbons (Table 1). Proton signals were all allocated by observation of HMQC correlations from δ_H_ 6.69 (1H, d, *J* = 9.6 Hz) to δ_C_ 120.2, δ_H_ 7.90 (1H, d, *J* = 9.6 Hz) to δ_C_ 140.7, δ_H_ 8.09 (1H, d, *J* = 2.0 Hz) to δ_C_ 149.6, δ_H_ 6.92 (1H, d, *J* = 2.0 Hz) to δ_C_ 110.0, δ_H_ 2.84 (2H, br d, *J* = 7.6 Hz) to δ_C_ 39.1, δ_H_ 4.78 (1H, tq, *J* = 7.6, 1.2 Hz) to δ_C_ 115.9, δ_H_ 1.53 (3H, d, *J* = 1.2 Hz) to δ_C_ 24.7, δ_H_ 1.42 (3H, d, *J* = 1.2 Hz) to δ_C_ 17.1, and δ_H_ 3.03 (3H, s) to δ_C_ 51.8. The above ^1^H- and ^13^C-NMR data were partly similar to those of alloimperatorin (**8**), which is a furocoumarin conjugated a prenyl unit [16]. Firstly, the proton signal of olefinic methine at δ_H_ 4.78 (tq, *J* = 7.6, 1.2 Hz) showed the same couple constants as two methyl protons (δ_H_ 1.53, d, *J* = 1.2 Hz and 1.42, d, *J* = 1.2 Hz) and methylene protons at δ_H_ 2.84 (br d, *J* = 7.6 Hz). HMBC spectra showed correlations from the olefinic triplet (δ_H_ 4.78) to the methyl resonances δ_C_ 24.7, from the methylene proton (δ_H_ 2.84) to δ_C_ 115.9 and 136.3, from the two methyl proton resonances (δ_H_ 1.53 and 1.42) to δ_C_ 115.9 and 136.3. The above data together with the ^1^H-^1^H COSY correlations between δ_H_ 4.78 and the methylene proton at δ_H_ 2.84 indicated the presence of a prenyl unit in **1**. Secondly, a pyrone ring residue of coumarin including resonances of δ_C_ 158.7, 120.2, 140.7, 150.9, 126.6 and δ_H_ 6.69, 7.90 could be identified by combining analysis of ^1^H-, ^13^C-NMR and HMBC spectra. In which, two coupling proton signals at δ_H_ 6.69 (1H, d, *J* = 9.6 Hz, H-3) and δ_H_ 7.90 (1H, d, *J* = 9.6 Hz, H-4) assigned to the double bond protons of pyrone ring presented HMBC correlations with δ_C_ 158.7 (C-2), 150.9 (C-9), and 126.6 (C-10). In addition, HMBC correlations from δ_H_ 8.09 (1H, d, *J* = 2.0 Hz) and 6.92 (1H, d, *J* = 2.0 Hz) to δ_C_ 138.4 and 147.3 suggested the presence of a furan ring which included resonances of δ_H_ 8.09 (H-2′), 6.92 (H-3′), δ_C_ 149.6 (C-2′), 110.0 (C-3′), 138.4 (C-6), and 147.3 (C-7). Interestingly, the methylene proton (δ_H_ 2.84) of prenyl was largely upfield shifted comparing with that of **8**, with chemical shift at δ_H_ 3.72 (2H, d, *J* = 7.2 Hz), indicating that the prenyl was no longer attached to an unsaturated carbon of the furocoumarin moiety. The observation of HMBC correlations from δ_H_ 2.84 to carbon signals at δ_C_ 76.4, 126.6, and 138.4 illustrated that the prenyl located at the quaternary carbon at δ_C_ 76.4 which was adjacent to the pyrone and furan ring. In addition, the resonance δ_C_ 164.7 at downfield shift in ^13^C-NMR spectrum could be assigned as a conjugated ketone carbon according with the presence of α, β-unsaturated carbonyl stretching vibration (ν_max_ 1687 cm^–1^) in IR spectrum. Long-rang HMBC correlations from H-4 (δ_H_ 7.90) to δ_C_ 164.7 suggested the adjacency relation between the pyrone ring and the ketone carbon. The location of the methoxyl substituent was confirmed by observing the long-range correlation from methoxyl proton signal at δ_H_3.03 (3H, s) to the quaternary carbon at δ_C_ 76.4. Thus, the chemical structure of **1** was finally elucidated as illustrated in Figure 1, and was named oxyalloimperatorin. The stereochemistry of C-5 remains to be determined.

**Table 1:** ^1^H-NMR and ^13^C-NMR data of compound **1** (Acetone-*d4*, 400 MHz).

Position	δ_C_, mult.	δ_H_ (*J* in Hz)
2	158.7, C	
3	120.2, CH	6.69 d (9.6)
4	140.7, CH	7.90 d (9.6)
5	76.4, C	
6	138.4, C	
7	147.3, C	
8	164.7, C	
9	150.9, C	
10	126.6, C	
2'	149.6, CH	8.09 d (2.0)
3'	110.0, CH	6.92 d (2.0)
1"	39.1, CH_2_	2.84 br d (7.6)
2"	115.9, CH	4.78 tq (7.6, 1.2)
3"	136.3, C	
4"	24.7, CH_3_	1.53 d (1.2)
5"	17.1, CH_3_	1.42 d (1.2)
5-OCH_3_	51.8, CH_3_	3.03 s

**Figure 2:**
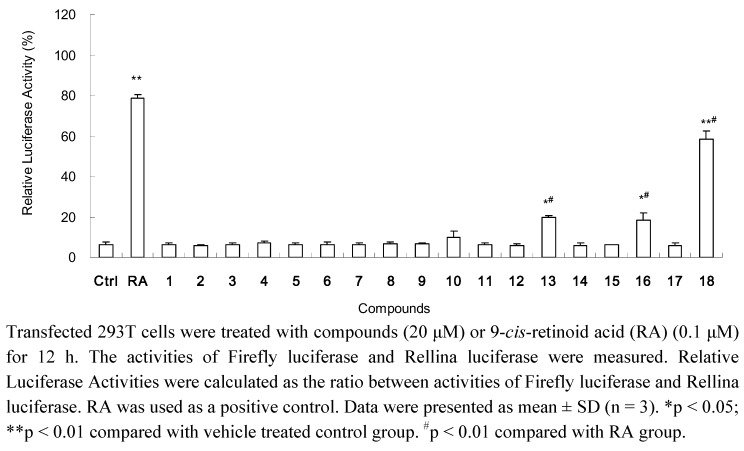
Promoting effects of isolated compounds on reporter transcription activities of RXRα.

All isolated compounds were furocoumarin skeleton derivatives. Their effects on gene transactivity of RXRα were evaluated by a Dual-Luciferase reporter assay system. It is well known that 9-*cis*-retinoid acid (9-*cis*-RA) could largely promote the reporter transcription [27]. As shown in Figure 2, consistent with previous results, treatment of cells with 9-*cis*-RA (0.1 μM) significantly (P < 0.01) induced the reporter transcription with a relative luciferase activity of 79%.

**Figure 3:**
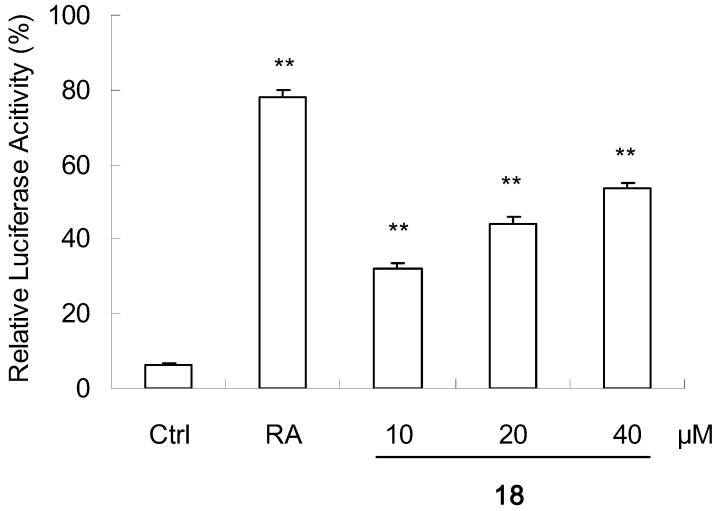
Promoting effect of compound **18** on reporter transcription activities of RXRα with concentration-dependence.

Among the isolated compounds, **18** (20 μM) significantly (P < 0.01) increased the transcriptional activation of RXRα, while **13** and **16** (each at 20 μM) showed weak effects (P < 0.05) on increasing the reporter transcription (Figure 2). Furthermore, **18** with concentration-dependent effect in the range of 10~40 μM is shown in Figure 3. 

**Figure 4:**
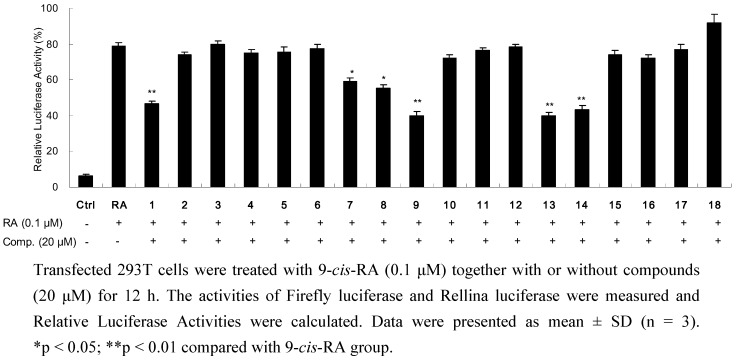
Inhibitory effects of isolated compounds on reporter transcription activities of RXRα.

**Figure 5:**
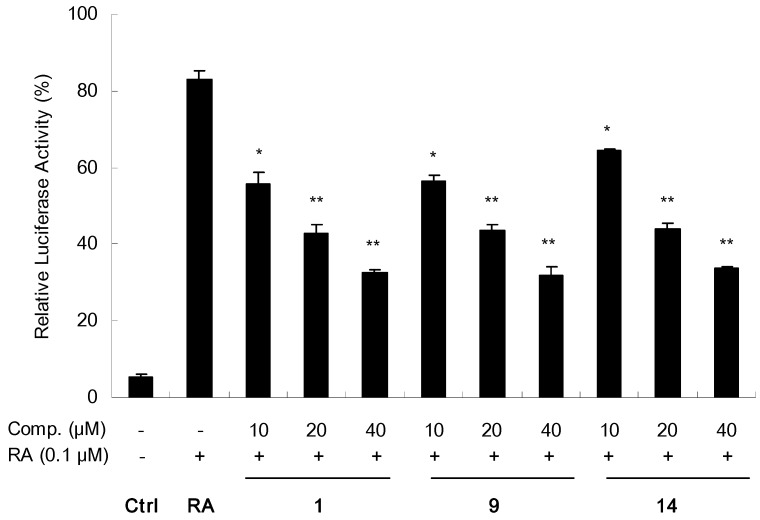
Inhibitory effects of compounds **1**, **9** and **14** on reporter transcription activities of RXRα in concentration-dependent manner.

All the isolated compounds were also examined for their possibility to inhibit the transactivity of RXRα using a similar assay as previous report, in which 9-*cis*-RA was simultaneously added to strongly induce RXRα gene transactivity [14]. Among tested compounds, **7**–**9**, **13**, **14**, and the new structure **1** (each at 20 μM) showed significant reduction in the relative luciferase activity induced by 9-*cis*-RA (Figure 4). In addition, **1**, **9** and **14** were further measured in three different concentrations at 10, 20 and 40 μM. As shown in Figure 5, all three metabolites exhibited good concentration-dependent inhibitory effects. 

The above findings indicate that these furocoumarin skeleton derivatives might have useful impact on many intractable diseases, such as cancer and metabolic diseases, due to their potential effects on regulating the transcriptional activation function of RXRα. Interestingly, compound **13** showed not only weakly increased the reporter transcriptional activation of RXRα but it also reduced the transactivity of RXRα induced by 9-*cis*-RA. One of the possible reasons could be that **13** and 9-*cis*-RA bind competitively with RXRα when they were simultaneously added to cells. Because of the transactive effect of **13** was rather weaker than that of 9-*cis*-RA (Figure 2), the transactivity of RXRα induced by 9-*cis*-RA was therefore decreased. However, this speculation and whether all the candidate compounds could bind to RXRα to regulate its transcriptional expression or not needs to be confirmed by further study.

## 3. Experimental

### 3.1. General

High-performance liquid chromatography (HPLC)-grade solvents were purchased from Merck KGaA (Darmstadt, Germany). Analytical reagents were obtained from Sinopharm Chemical Reagent co., Ltd (Shanghai, China). Silica gel (200–300 mesh) used in column chromatography and TLC plates were bought from Qingdao Haiyang Chemical Co., Ltd (Qingdao, China). YMC gel ODS-A was purchased from YMC co., Ltd (Allentown, USA). NMR spectra were recorded on a Bruker Avance 400 spectrometer using tetramethylsilane as the internal reference. HR-ESI-MS were recorded on Bruker FT-MS. IR spectrum was carried on a Nicolet IR200 (Thermo Electron Corporation, U.S.A.). Rotation data was obtained from a 341 polarimeter (PerkinElmer Co. Ltd. U.S.A). The values of luciferases were measured on a 1420 VICTOR^3^_TM_ V (PerkinElmer, Boston, MA, USA).

### 3.2. Materials

Radix Angelicae Dahuricae, the dry root of A. *dahurica* (Fisch. ex Hoffm) Benth, et Hook. f, was purchased from a store of Tongrentang Pharmacy (Hangzhou City, Zhejiang Province, China) and identified by Mrs. Xiuhong Zhou (Senior Engineer, Forestry Bureau of Yongchun, Quanzhou City, China). A voucher specimen was deposited at the School of Pharmaceutical Sciences in Xiamen University, Xiamen, China. Plasmids (pBind RXRα LBD and pG5 luc) were provided by Dr. Xiao-kun Zhang from the Burnham Institute for Medical Research, Cancer Center, La Jolla, CA, USA. Dual-Luciferase Reporter Assay System Kit was purchased from Promega Corporation. Lipofectamine 2000 reagent was bought from Invitrogen Co., Ltd.

### 3.3. Extraction and Isolation

The dried root of *A. dahurica* (3 kg) was boiled and refluxed for 2 h with 60% of aqueous ethanol solution (5 L × 3 times). After filtration, the extracted solution was concentrated *in vacuo*. The condensate was then suspended in H_2_O (5 L) and partitioned with EtOAc (5 L × 3 times). The EtOAc extracts were combined and evaporated under vacuum to afford an EtOAc-soluble extract (48.3 g). The EtOAc-soluble extract (45.0 g) was chromatographed on silica gel column using stepwise gradient elution with CHCl_3_-MeOH (100:0~0:100) to obtain 11 fractions (Fr. 1-11). Fr.2 (12.1 g) was subjected to silica gel column chromatography eluting with *n*-hexane-EtOAc (98:2~1:1) to get 10 subfractions (Fr.2-1 ~ Fr.2-10). Fr.2-5 (1.9 g) was applied to YMC ODS column chromatography and eluted with aqueous acetonitrile (55~100%) to give compound **2** (257.0 mg). Fr.2-6 (1.3 g) was subjected to YMC gel ODS column chromatography and eluted with aqueous methanol (40~100%) to give **3** (14.0 mg). Fr.2-7 (2.0 g) was chromatographed on YMC gel ODS-A column and eluted with aqueous methanol (50~100%) to give **4** (300 mg) and a subfraction (Fr.2-7-2) which was further purified through preparative HPLC (Restek Prinnacle DB C18, 5 μm, 250×10 mm) eluting with 60% of aqueous methanol solution to afford **5** (40.0 mg, Rt 14.0 min) and **6** (61.0 mg, Rt 17.5 min). Fr.2-8 (782.0 mg) was subjected to YMC gel column chromatography using the elution of aqueous methanol (30~100%) to yield **7** (13.0 mg). Fr.2-9 (738.0 mg) was applied to YMC gel column chromatography and eluted with aqueous methanol solution (40~100%) to produce **8** (58.0 mg) and a subfraction (Fr.2-9-2) which was further purified through preparative HPLC eluting with MeOH-H_2_O (60:40) to afford **9** (39.0 mg, Rt 6.5 min) and **10** (23.0 mg, Rt 10.5 min). Fr.2-10 (883.0 mg) was subjected to YMC gel column and eluted with aqueous methanol solution (40~100%) to get **11** (44.0 mg), **12** (21.0 mg), **1** (12.0 mg), and subfraction Fr.2-10-4 (100 mg) was chromatographed on YMC gel to get **13** (14.0 mg) and Fr.2-10-4-1 which was further purified through preparative HPLC eluting with MeOH-H_2_O (70:30) to afford **14** (29.0 mg, Rt 6.5 min) and **15** (38.0 mg, Rt 7.5 min). Fr.4 (2.5 g) was applied to YMC gel column chromatography with aqueous methanol solution (40~100%) as elution to afford **16** (256.0 mg). Fr.5 (2.0 g) was subjected to silica gel column eluted with EtOAc-MeOH (96:4~0:100) to afford Fr.5-7(155 mg) which was crystallized in chloroform to obtain **17** (50.0 mg). Fr.7 (5.18 g) was subjected to silica gel column chromatography eluted with CHCl_3_-MeOH (99:1~0:100) to afford Fr.7-3. The subfraction (0.8 g) was applied to silica gel column and eluted with CHCl_3_-Acetone (95:5~0:100) to afford **18** (131.0 mg).

*Oxyalloimperatorin* (**1**): white, amorphous powder, [α]^22^_D _ +5 (*c* 0.4, MeOH). UV (MeOH) λ_max_ 361 (log *ε* 3.50) nm; IR (MeOH) ν_max_ 2923, 1738, 1687, 1625, 1438, 1082 cm^–1^; ^1^H-NMR (400 MHz, acetone-*d4*) and ^13^C-NMR (100 MHz, acetone-*d4*), see Table 1; HR-ESI-MS *m*/*z* 323.0894 [M + Na]^+^ (calcd for C_17_H_16_O_5_, 323.0890), *m*/*z* 339.0643 [M + K]^+^ (calcd 339.0629). 

### 3.4. Cell Culture and Dual-Luciferase Reporter Gene Assay

Human embryonic kidney 293T cells were cultured in DMEM medium containing 10% fetal bovine serum (FBS). The previous dual-luciferase reporter gene assay with some modification was used in the present study [14,27]. In brief, approximately 4 × 10^4^ cells/well were seeded in 24-well plates. Cells were transfected with two plasmids, 30 ng pBind RXRα LBD and 60 ng pG5 luc using Lipofectamine 2000 (Invitrogen). After 24 h, cells were exposed to tested compounds for 12 h. Then, the cells were washed with PBS and lysed with passive lysis buffer (1 × PLB) on rocking platform for 15 min. The activities of Firefly luciferase and Rellina luciferase were examined according to the introduction of Dual-Luciferase Reporter Assay System Kit. Relative luciferase activities were obtained as the ratio between activities of Firefly luciferase and Rellina luciferase.

### 3.5. Statistical Analysis

The results were expressed as mean ± standard diviation (S.D.) from at least three independent experiments. Statistical significances were compared between two groups. Statistical analysis was performed with the Student’s t-test. The value of *P* < 0.05 was considered statistically significant.

## 4. Conclusions

One novel furocoumarin derivative **1**, together with seventeen furocoumarins **2–18** were isolated from the radix of *Angelica dahurica*. The chemical structure of new metabolite was characterized by analysis of IR, NMR, and HR-ESI-MS spectroscopic data. Among these furocoumarin skeleton derivatives, the new compound **1**, and known compounds **7–9**, **13**, **14**, **16** and **18** showed the potential activities in regulating transcriptional activation function of RXRα. These metabolites might show beneficial effects against intractable diseases with relation to RXRα, for example anti-cancer and anti-diabetes. The various bioactivities of these metabolites and their molecular mechanism of action relating to nuclear receptor RXRα could be examined in the future study.
